# Zona pellucida from fertilised human oocytes induces a voltage-dependent calcium influx and the acrosome reaction in spermatozoa, but cannot be penetrated by sperm

**DOI:** 10.1186/1471-213X-6-59

**Published:** 2006-12-05

**Authors:** Catherine Patrat, Jana Auer, Patricia Fauque, Roger L Leandri, Pierre Jouannet, Catherine Serres

**Affiliations:** 1 Biologie de la Reproduction, Hôpital Cochin, AP-HP, Université Paris Descartes, Faculté de Médecine, Paris, France

## Abstract

**Background:**

The functions of three zona glycoproteins, ZP1, ZP2 and ZP3 during the sperm-zona pellucida (ZP) interaction are now well established in mice. The expression of an additional zona glycoprotein, ZPB/4, in humans, led us to reconsider the classical mouse model of gamete interaction. We investigated the various functions of human ZP (hZP) during the interaction of spermatozoa with fertilised and unfertilised oocytes.

**Results:**

The hZP of fertilised oocytes retained their ability to bind sperm (albeit less strongly than that from unfertilised oocytes), to induce an intraspermatic calcium influx through voltage-dependent channels similar to that observed with hZP from unfertilised oocytes and to promote the acrosome reaction at a rate similar to that induced by the ZP of unfertilised oocytes (61.6 ± 6.2% vs60.7 ± 9.1% respectively). Conversely, the rate of hZP penetrated by sperm was much lower for fertilised than for unfertilised oocytes (19% vs 57% respectively, p < 0.01). We investigated the status of ZP2 in the oocytes used in the functional tests, and demonstrated that sperm binding and acrosome reaction induction, but not ZP penetration, occurred whether or not ZP2 was cleaved.

**Conclusion:**

The change in ZP function induced by fertilisation could be different in human and mouse species. Our results suggest a zona blocking to polyspermy based at the sperm penetration level in humans.

## Background

Sperm interaction with the extracellular matrix surrounding the oocyte, the zona pellucida (ZP), is a key step in mammalian fertilisation. The structure and function of the ZP were first studied in mice in the 1980s [1-3]. Mouse ZP contains three glycoproteins: ZP1, ZP2 and ZP3. ZP3 binds to primary receptors on capacitated spermatozoa, inducing a cascade of intraspermatic events including the biphasic calcium influx leading to the acrosome reaction (AR). Following the AR, spermatozoa remain bound to the ZP via ZP2; they then penetrate the ZP and fuse with the egg plasma membrane [4]. The fertilisation is followed by the fusion of peripheral cortical granules with the vitelline membrane, resulting in the discharge of the contents of these granules into the perivitelline space. This exocytosis modifies the ZP matrix such that sperm no longer bind to or penetrate ZP, preventing polyspermy.

Our understanding at a molecular level of gamete interaction in humans is much more restricted than for mice and other mammalian species, due to the small number of oocytes available which limits the number of feasible studies. In humans, only a few studies with recombinant human ZP3 (rhZP3) have been carried out, mainly on the AR and its regulation by ions [5,6]. Moreover, the probable structural differences between natural ZP and rhZP3 – particularly in terms of the carbohydrate moieties which play an important role in the recognition and binding of the ZP by sperm [7] – raise some doubt as to whether rhZP precisely mimics natural hZP in its action. Therefore some data obtained in mice have been extrapolated to humans. However, studies of genes encoding ZP proteins led to the recent discovery of a fourth zona glycoprotein in humans; this protein is not present in mice but is found in other mammalian species [8,9]. This fourth zona glycoprotein, ZP4, plays a structural role and is also involved, together with ZP3, in sperm binding and AR induction [10-13]. The identification of this fourth glycoprotein raises the possibility that classical model of sperm-ZP interaction established in mice may not apply to species producing ZP4. The functions of the ZP should therefore be re-evaluated in such species.

The aim of our study was to analyse the functions of human ZP (hZP) from fertilised and unfertilised oocytes, obtained from an In Vitro Fertilisation (IVF) program, during their interaction with spermatozoa. We investigated the capacity of hZP to bind spermatozoa, to induce calcium signalling and the AR. We analysed in detail the intracellular calcium signalling induced by the hZP as most of the knowledge of this ZP-induced transduction pathway comes from studies carried out in cattle and mice [14,15] We also assessed the penetration of hZP by spermatozoa, as a means of evaluating hZP function during secondary binding.

## Results

### Biochemical characterisation of ZP collected from unfertilised and fertilised human oocytes

**Figure 1:**
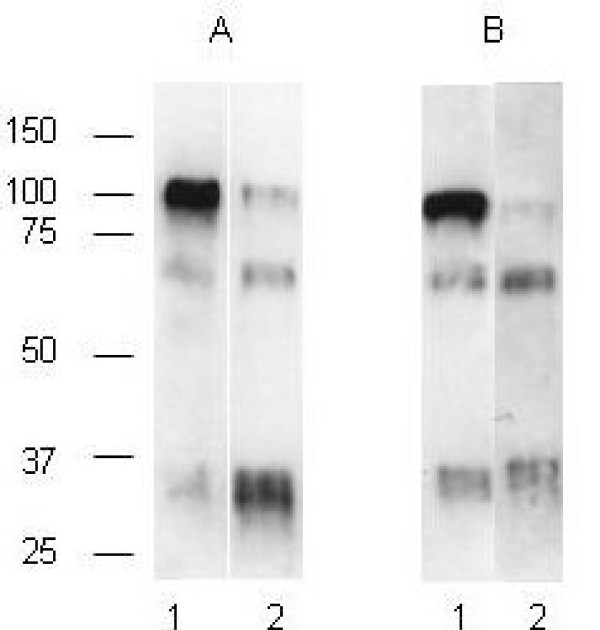
**Biochemical characterisation of hZP2 from unfertilised and fertilised oocytes**. ZP were isolated from a pool of 3 unfertilised (lane 1) and 3 fertilised (lane2) oocytes recovered after an IVF procedure (A) or a sperm-binding experiment (B) (experiment 1, Table 1). Oocytes were cleared of bound spermatozoa before ZP isolation. ZP proteins were then solubilised in Laemmli buffer and separated by SDS-PAGE in a 7% polyacrylamide gel. Glycoproteins were transferred to a PVDF membrane and ZP2 was detected by incubation with anti-rhZP2 and enhanced chemiluminescence. Molecular masses are indicated on the left.

In addition to morphological criteria, biochemical analyses on the ZP was performed to categorise the oocytes as "fertilised" or "unfertilised". As proteolysis of ZP2 is a known biochemical modification of the ZP occurring after the fertilisation, we determined by western blotting the cleavage status of ZP2 in ZP isolated from the unfertilised and fertilised oocytes used in our work (Figure 1). In unfertilised embryos, the anti-rhZP2 antibody detected the ZP2 as a major band at 100 kDa (lane 1 in Figure 1A and 1B), which disappeared almost totally in fertilised oocytes and was essentially replaced by two bands of 70 kDa and 30 kDa (lane 2 in Figure 1A and 1B). This result indicates that our hZP preparations were suitable to represent a fertilised and unfertilised human oocyte/egg material.

### Sperm binding to intact hZP from fertilised and unfertilised human oocytes

**Figure 2:**
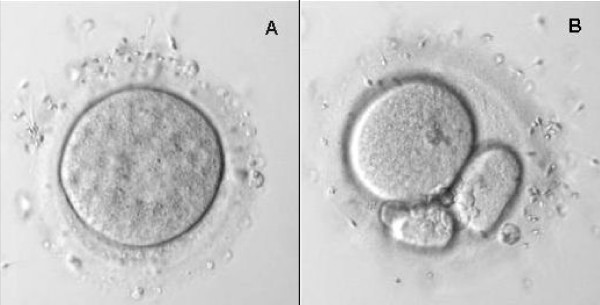
**Sperm binding test using unfertilised and fertilised human oocytes**. Unfertilised human oocytes recovered after IVF failure (A) and fertilised oocytes with a high fragmentation rate two days after fertilisation (B), were cleared of bound spermatozoa and re-inseminated. After three hours, oocytes were similarly washed by repeated aspiration in a pipette, and the number of spermatozoa bound to the surface was determined under a microscope. Original magnification ×400.

After 3 h hours of gamete co-incubation, the zonae pellucidae of all inseminated oocytes, whether fertilised or not, were able to bind spermatozoa (Figure 2). The number of spermatozoa bound by intact ZP varied from 12 to 76 for unfertilised and from 6 to 65 for fertilised oocytes. However, the mean number of spermatozoa bound *per *fertilised oocyte was significantly lower compared to unfertilised oocyte (p < 0.05, Table 1).

**Table 1:** Sperm binding to intact zona of unfertilised and fertilised human oocytes

Experiment	Unfertilised oocytes	Fertilised oocytes
1	14.5 ± 3.3 (12)^a^	15.5 ± 3.7 (10)
2	20.6 ± 6.0 (10)	8.4 ± 1.5* (21)
3	46.8 ± 9.1 (9)	29.7 ± 5.0* (12)

Results are expressed as the number of spermatozoa bound per oocyte (mean ± SEM); ()^a ^number of oocytes ; * significantly different from unfertilised oocytes (Mann and Whitney test; p < 0.05)

### Ability of solubilised ZP from fertilised and unfertilised human oocytes to induce calcium influx into capacitated spermatozoa

#### Description and quantification of the hZP-induced Ca^2+ ^influx

**Figure 3:**
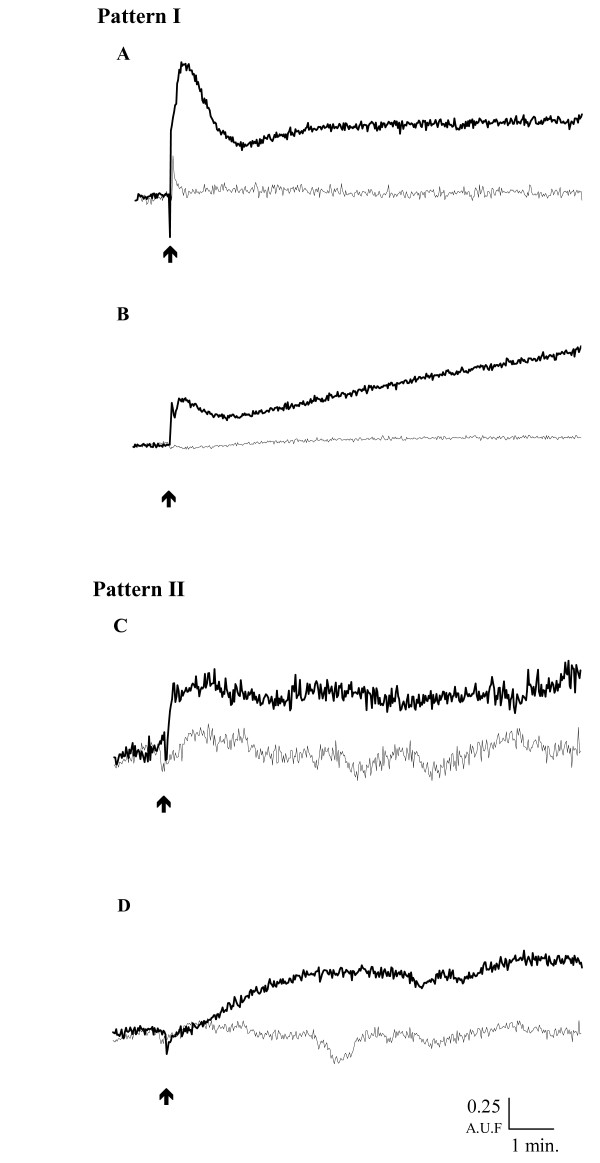
**Time-course of the intracellular Ca^2+ ^modifications induced by human zona pellucida in spermatozoa**. Capacitated, Fura-2-loaded spermatozoa (15 × 10^6^/ml) were incubated at 37°C in BWW medium. After stabilisation of the fluorescent signal, solubilised hZP (2/μl final concentration) (heavy line) or the same volume of solvent (light line) was added to the sperm suspension (arrow). Results are expressed as the fluorescence excitation ratio (340/380 excitation wavelengths, in arbitrary fluorescence units (AFU)) as a function of time (min). Two patterns of change in calcium concentration were observed in response to the addition of hZP to sperm samples: biphasic (Pattern I A and B) or monophasic (Pattern II C and D).

Regardless of the origin of the hZP, we analysed sperm calcium response to hZP in all the experiments (n = 21). The addition of solubilised hZP (2 hZP/μl) to the capacitated human sperm samples induced 2 types of intraspermatic increase in Ca^2+ ^concentration. In 12 of the 21 samples, solubilised hZP induced a biphasic Ca^2+ ^influx consisting of a transient peak followed by a sustained increase in [Ca^2+^] above basal levels called pattern I (Figure 3A and 3B). In pattern I, the transient peak lasted 30 to 170 s and corresponded to an increase in calcium concentration of 775.4 ± 190.8 nM (Table 2). In pattern I, the second sustained influx of 644.2 ± 215.4 nM Ca^2+ ^stabilised at a level below (n = 6/12, Figure 3A) or above (n = 6/12, Figure 3B) the initial peak, 2 to 15 min after the addition of hZP. No significant influx was observed in control experiments (Figure 3 and Table 2). In the other nine experiments, hZP induced only a sustained calcium influx called pattern II (Figures 3C and 3D), similar to the second phase of pattern I. In pattern II, the maximal level was reached rapidly (mean of 32.5 ± 5.6 s) in four experiments (Figure 3C) and more slowly (mean of 443.4 ± 32.2 s) in the other five experiments (Figure 3D). In the pattern II, mean [Ca^2+^]_i _during the sustained phase was also significantly higher than in the controls, reaching values similar to those observed in the sustained phase of pattern I (Table 2).

**Table 2:** Effect of solubilised human zona pellucida on intracellular Ca^2+ ^concentration and the acrosome reaction (AR) of capacitated spermatozoa

		[Ca^2+^]_i _(nM)			Ca^2+ ^influx(nM)		AR	
				
Type of [Ca^2+^] increase	Treatment	Basal level	Peak	Sustained phase	Peak^b^	Sustained phase^c^	(%)	ZPIAR^d ^(%)
Pattern I (n = 12)	hZP^a^	386.7 ± 35.2	1162.1 ± 196.5***	1030.8 ± 232.4**	775.4 ± 190.8***	644.2 ± 215.4^**^	61.4 ± 7.7^*^	44.0 ± 6.5
	Control	345.8 ± 42.5	363.7 ± 39.3	380.8 ± 33.2	17.9 ± 15.9	35.0 ± 15.5	17.4 ± 2.1	

Pattern II (n = 9)	hZP^a^	487.4 ± 62.7		1188.9 ± 230.2**		701.4 ± 178.3**	60.9 ± 6.6*	34.9 ± 5.8
	Control	441.1 ± 42.9		467.8 ± 52.0		26.7 ± 25.0	26.0 ± 2.6	

All the values are expressed as means ± SEM; the number of experiments is shown in brackets (n=); [Ca^2+^]_i _= intracellular Ca^2+ ^concentration; AR = acrosome reaction; pattern I corresponds to a biphasic increase in [Ca^2+^]_I_; pattern II corresponds to a monophasic increase in [Ca^2+^]_i _; ^a^2 ZP/μl were incubated with the spermatozoa; ^b^Δ between [Ca^2+^]_i _at peak and basal level; ^c^Δ between [Ca^2+^]_i _at sustained phase and basal level; ^d^human zona pellucida-induced acrosome reaction; *,**,*** statistically different from respective control *p < 0.05,**p < 0.01,***p < 0.001 with Student's t test.

The data of 21 experiments presented in Table 2 were re-analysed as a function of hZP origin in Table 3. Solubilised hZP from fertilised oocytes induced a calcium response in capacitated spermatozoa, with a similar amplitude or pattern to that obtained with hZP from unfertilised oocytes.

**Table 3:** Effect of solubilised  human zona pellucida providing from fertilised and unfertilised oocytes on sperm Ca^2+ ^concentration and acrosome reaction rate

		**[Ca^2+^] increase pattern **^a^		**[Ca^2+^]_i _(nM)**			
				
**Oocytes**	**Treatment**	I	II	Basal level	Peak^b^	Sustained phase	**AR (%)**
**Unfertilised (n = 9)**	hZP	4	5	448.5 ± 50.4	1012.2 ± 229.7*	1171.7 ± 262.4*	60.7 ± 9.1**
	Control			383.3 ± 44.1	423.3 ± 41.8	448.8 ± 49.2	21.8 ± 2.7

**Fertilised (n = 12)**	hZP	8	4	416.0 ± 48.5	902.5 ± 165.9**	1050.4 ± 217.4*	61.6 ± 6.2***
	Control			389.2 ± 45.9	378.7 ± 46.6	395.0 ± 38.0	20.7 ± 2.7

The results are expressed as mean ± SEM; The number of experiment is shown in brackets (n =); [Ca^2+^]_I _= intracellular Ca^2+ ^concentration; AR = acrosome reaction^a ^Number of sperm samples displaying pattern I or II increases in calcium concentration, as shown in Figure 1; ^b ^peak was calculated only for sperm samples displaying pattern 1; *, **, *** statistically different from the corresponding control: * p < 0.05, ** p < 0.01,*** p < 0.001 with Student's t test.

#### Involvement of a voltage-dependent channel in the increase in calcium concentration induced by solubilised human zona pellucida

**Figure 4:**
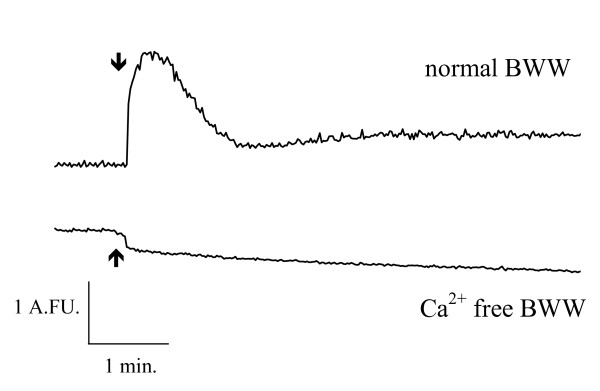
**Effect of extracellular Ca^2+ ^depletion on the human zona pellucida-induced intracellular [Ca^2+^] increase in spermatozoa**. Capacitated, Fura-2-loaded spermatozoa were incubated in normal BWW medium (upper curve) or in BWW medium lacking calcium (lower curve). One minute before adding hZP, EGTA (500 μM) was added to the Ca^2+ ^free BWW, to ensure that the extracellular medium was devoid of Ca^2+^. Solubilised hZP (2/μl) was added (arrow) to the medium for each incubation. Results are expressed as the fluorescence excitation ratio (340/380 excitation wavelengths, in arbitrary fluorescence units (AFU)) as a function of time (min). A typical experiment is shown.

**Figure 5:**
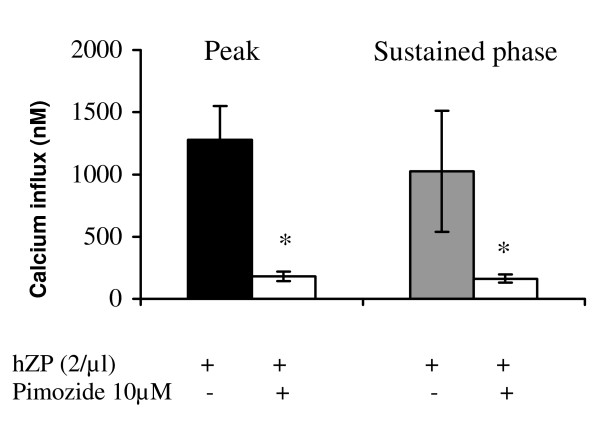
**Effects of pimozide on the Ca^2+ ^influx stimulated by hZP**. Spermatozoa were capacitated in the presence or absence of 10 μM pimozide. They were then loaded with Fura-2 in BWW medium and hZP (2/μl) was added (n = 3 experiments). The results are expressed as the mean ± SEM of Ca^2+ ^influx induced by hZP, for the peak and the sustained phase; *p < 0.001, Mann and Whitney test.

In the absence of external Ca^2+^, the addition of hZP did not induce an influx of Ca^2+ ^(Figure 4). The effect of pimozide, which efficiently blocks Ca^2+ ^currents induced by solubilised ZP in mouse spermatozoa when added at the start of capacitation [16], was investigated on the calcium response to solubilised hZP. The incubation of sperm samples with 10 μM pimozide from the start of capacitation resulted in 91.7 ± 4.8% inhibition of the solubilised hZP-stimulated Ca^2+ ^peak and 81.2 ± 11.8% inhibition of the sustained phase (p < 0.05, n = 3; Figure 5).

### Ability of ZP from fertilised and unfertilised human oocytes to induce AR in capacitated spermatozoa

#### Induction of the acrosome reaction by solubilised ZP

When spermatozoa were incubated with hZP there was a significantly higher proportion of AR than in the absence of hZP (61.2 ± 5.1% vs 21.1 ± 1.9%, respectively, n =.21, p < 0.001). The mean percentage of ZP-induced AR did not differ significantly between the sperm populations displaying pattern I and II increases in calcium levels (44.0 ± 6.5% vs 34.9 ± 5.8%, respectively, Table 2). No correlation was found between ZPIAR (%) and basal calcium level (r^2 ^= 0.17), hZP-induced calcium peak (r^2 ^= 0.01) or sustained calcium phase (r^2 ^= 0.06).

The mean AR rate induced by solubilised ZP from fertilised oocytes differed significantly from the spontaneous AR rate (61.6 ± 6.2% vs 20.7 ± 2.7% respectively, p < 0.001) but not from that induced by solubilised hZP from unfertilised oocytes (Table 3).

#### Induction of the acrosome reaction by intact ZP

In 3 independent experiments, we found that most of the spermatozoa bound to intact zona had undergone the AR, whether the oocytes were fertilised or unfertilised. After a three-hour co-incubation period, the mean rate of the AR in spermatozoa bound to fertilised oocytes was significantly higher than that of free-swimming spermatozoa in the medium (mean +/- SEM: 59.0 ± 16.2% vs 11.7 ± 3.5% respectively, p < 0.001, n = 3) but did not differ from that induced by ZP from unfertilised oocytes (63.0 ± 9.3%). After 18 h of co-incubation, the frequency of the AR had increased slightly in zona-bound spermatozoa but remained similar in unfertilised and fertilised oocytes (70.7 ± 2.5% and 85.0 ± 6.0%, respectively, n = 3).

### Sperm penetration in intact human zona pellucida from fertilised and unfertilised oocytes

The mean number of embedded spermatozoa was significantly higher after co-incubation of spermatozoa with unfertilised intact hZP compared to fertilised hZP (p < 0.001; Table 4). The penetration rate (proportion of penetrated hZP) was significantly higher for unfertilised than for fertilised oocytes (p < 0.05, Table 4) in each of the 3 experiments. The overall penetration rate for unfertilised and fertilised oocytes was 57% and 19%, respectively.

**Table 4:** Human sperm penetration into the ZP of unfertilised and fertilised oocytes

	Unfertilised oocytes		Fertilised oocytes	
	
Experiment	Number of embedded spermatozoa per ZP mean ± SEM	Penetrated zona pellucida %	Number of embedded spermatozoa per ZP mean ± SEM	Penetrated zona pellucida %
1	0.93 ± 0.32	57 (8/14)^a^	0.18 ± 0.13**	18* (2/11)
2	1.14 ± 0.60	57 (4/7)	0.22 ± 0.11**	17*(4/23)
3	1.14 ± 0.43	57 (8/14)	0.26 ± 0.13**	21*(4/19)

Total	1.06 ± 0.23	57 (20/35)	0.23 ± 0.07***	19*** (10/53)

()^a^number of oocytes with penetrated zona pellucida/total number of oocytes; *,**,*** statistically different from unfertilised oocytes: * p < 0.05; ** p < 0.01; *** p < 0.001 with Student's t test (mean ± SEM) or Chi-squared test (%).

## Discussion

Most of our knowledge about the events occurring during gamete interaction has come from studies carried out in mice. A model rapidly emerged, with each of the three zona glycoproteins having a precise function. ZP1 was thought to be purely structural, with dimers creating bridges between filaments composed of a succession of ZP2 and ZP3 molecules. ZP2 and ZP3 were shown to interact directly with sperm membrane receptors after and before the AR, respectively [4]. The demonstration that many species, including humans, contain a fourth zona protein, ZP4, which is absent in mice, raises questions about the extent to which the murine model can be applied to other ZP4-containing species. Our study characterises the human ZP bioactivity before and after fertilisation. To reach this objective, we have collected both unfertilised and fertilised human oocytes and the physiological relevance of this material to real unfertilised oocytes or fertilised zygotes must be considered.

### Relevance of unfertilised and fertilised oocytes to study ZP bioactivity

For practical and ethical reasons, only unfertilised or fertilised oocytes which are usually discarded in an IVF program can be used for experiments. Consequently, the oocytes and zygotes have to be cultured for 48 h before deciding to use them for research. Could this condition be responsible for uncertain or dubious physiological relevance of measured ZP bioactivity? It has been shown previously that unfertilised oocytes collected after IVF failure could be used to study sperm – ZP interaction [17]. In our study, unfertilised oocytes were mature but no pronucleus was observed 18 hours post insemination and no cleavage occurred within 48 hours. Furthermore, we controlled that there was no ZP2 proteolysis in these unfertilised oocytes. On the contrary, fertilised oocytes defined by the presence of at least 3 PN at 18 hours after fertilisation or by the presence of 2 PN but abnormal cleavage after 48 hours had a ZP2 cleaved in two bands of 70 and 30 kDa, denoting a limited proteolysis likely due to cortical granule exocytosis [18]. Therefore, we are confident that the material used in our study was relevant to unfertilised oocytes or fertilised zygotes to analyse hZP bioactivity.

### Fertilised human ZP may bind spermatozoa and induce acrosome reactions but are not penetrated by sperm

In mice, spermatozoa do not bind to fertilised oocytes and the ZP3 protein purified from two-cell embryos cannot induce the AR in mouse spermatozoa [4]. Surprisingly, we observed that zona pellucida of human fertilised oocytes, retain its capacity to bind spermatozoa, albeit about half as efficiently as zona from unfertilised oocytes and was able to induce an intra-spermatic calcium influx of the same amplitude as hZP from unfertilised oocytes. The spermatozoa bound to fertilised oocytes were found to be both acrosome-reacted and acrosome-intact spermatozoa. A recent study has reported that acrosome-reacted spermatozoa have a drastically reduced or zero ability to directly bind ZP [19]. Therefore, it may be hypothesised that acrosome-reacted spermatozoa observed on fertilised ZP were acrosome intact before attachment to ZP, and underwent the AR upon contact with hZP. Our data suggest that (1) hZP of fertilised oocytes is able to bind sperm and to induce AR (also confirmed by test using solubilised ZP of fertilised oocytes) and (2) acrosome-reacted spermatozoa remain attached to fertilised ZP despite the cleavage of ZP2. Thus the human glycoproteins involved in sperm binding and AR induction – presumably ZP4 (ZPB) and ZP3 (ZPC) [12,13] – seem to remain functional after fertilisation. This is the original point of our study that contrasts with observations in mice showing that fertilisation modifies the ZP, preventing sperm binding and AR induction. The ZP of mouse embryos was clearly shown to be unable to induce the AR in mouse spermatozoa, in experiments using purified ZP3 from the embryo as an inducer [3], but post-fertilisation modifications of mouse ZP3 have not been demonstrated directly. Thus, the mouse gamete interaction does not seem to model the situation in human in a reliable manner. Could the additional glycoprotein, ZP4, present in human oocyte but not in mice account for this difference? Does ZP4 work individually or in a heterocomplex with ZP3, as described in boars [10]?

In our study, the biochemical analysis of the hZP collected after fertilisation confirmed that ZP2 was cleaved, demonstrating that sperm binding and the AR can occur whether ZP2 is cleaved or uncleaved. Using a model of chimeric human-mouse oocytes obtained by transgenesis, Rankin et al. [20] suggested that sperm binding is a function of the supramolecular structure of the ZP, modulated by the cleavage status of ZP2, with sperm binding occurring when ZP2 is uncleaved but not when ZP2 is cleaved. Our observation of sperm binding to the zona pellucida when human fertilised oocytes with cleaved ZP2 were re-inseminated suggests that Rankin's model, although appropriate for oocytes with three zona glycoproteins as in mouse or chimeric human-mouse ZP, would not be applicable to species with four zona proteins, as in humans.

Intact hZP from fertilised and unfertilised human oocytes are equally efficient in inducing the AR, with similar rates as reported previously for unfertilised oocytes [21]. By contrast, solubilised ZP-induced AR rates measured in the present study were two times higher (ΔAR = 40%) than those previously reported (ΔAR = 20%) [22,23]. This difference can arise from different experimental conditions (time of capacitation, incubation medium...) or from the probes used to reveal the AR. We used the anti-CD46 antibody coupled to the flow cytometry to measure solubilised hZP-induced AR and not the PSA lectin labelling widely used in the other studies. From our knowledge, this is the first time that the flow cytometry, an objective method which allows counting on several thousand cells, was used to measure ZP-induced AR. As PSA lectin is not the most suitable probe for AR measurement in flow cytometry [24], we have chosen anti-CD46 antibody that gives for acrosome-reacted cells a high fluorescent peak well separated from the low fluorescent peak of intact cells [25]. By contrast, the fluorescent peaks of intact and acrosome-reacted spermatozoa are more or less overlapped when PSA lectin staining is used [26]. In our study, the use of two different probes to mark the AR induced by either solubilised (anti-CD46) or intact (PSA lectin) ZP, does not make it possible to compare the results. Indeed, it is known that different methods of AR staining never give the same results when the probes target different sites in the sperm cells, measuring different steps in the AR kinetics [27]. Our objective was to compare the AR inducing ability of ZP of fertilised and unfertilised oocytes within a same experiment.

In our experiments, more than 80% of fertilised oocytes co-incubated with capacitated spermatozoa had no spermatozoa within their ZP. This observation shows that human fertilised oocytes cannot be more penetrated by spermatozoa and is consistent with the murine model as demonstrated in previous studies [4,28]. This inhibition of sperm penetration could be temporally correlated with the proteolytic cleavage of ZP2, demonstrated after fertilisation in mice [29] and in humans [our study].

In summary, the events of ZP sperm binding/AR induction and zona penetration can be dissociated in humans. The main step of polyspermy blocking would be the inhibition of the acrosome-reacted sperm penetration through the zona after fertilisation. Some post-fertilisation architectural alterations could provoke a hardening of the zona which make it impossible to be lysed by the acrosomal enzymes.

### ZP-induced calcium influx in human capacitated spermatozoa is biphasic and involves voltage-dependent channels

Among the different functions of ZP analysed in this work, we have brought particular attention to the ZP-induced calcium influx in spermatozoa. Until now, this function of ZP had not been studied in humans, except with recombinant ZP3 as the inducer [5,6]. Our study brings detailed information on intraspermatic calcium increase induced by solubilised hZP. When disaggregated by acid treatment, it retained its ability to induce an intracellular increase in calcium concentration, as previously reported for AR induction [30-32].

The calcium influx induced by solubilised hZP displayed qualitative and quantitative aspects similar to those reported for other species. Using spectrofluorimetry, we found that the calcium response to hZP may be biphasic or monophasic, depending on the sample. Previous studies using spectrofluorimetry and a similar probe showed that the Ca^2+ ^response to solubilised mouse ZP [33] or recombinant hZP3 [5,6] was biphasic, with a first transient peak and a second sustained phase. However, in nine of the 21 cases analysed in our study, the calcium response appeared to be monophasic, with only sustained increase in calcium concentration. Either the transient calcium peak did not occur or it developed so rapidly that it was not recorded in our conditions. If the initial peak lasted 200 ms as in mice [16], the peak development was probably too rapid to be detected, and this could explain the previous reports of a monophasic increase in calcium concentration in response to ZP in cattle [34] and in mice [14,35]. However, Shirakawa and Miyazaki [36] detected no transient calcium peak with recordings taken at 0.3-second intervals in the hamster sperm head after the addition of solubilised ZP. These results and ours suggest that a sustained calcium influx, keeping [Ca^2+^]_i _high for a sufficiently long period, may be sufficient to trigger the AR. The importance of the sustained phase in AR induction is also illustrated by the similar AR rates induced by hZP in sperm samples with monophasic and biphasic calcium responses.

The calcium response to solubilised human ZP is sensitive to pimozide (>80% inhibition), suggesting that VOCC is involved in this ionic event. T-type VOCC activity, involving the Cay3.2 channel in particular, has been associated with AR in humans [37,38] and it has been shown that ZP-induced calcium entry and AR depend on T-type VOCC in mice [39].

## Conclusion

One of the major findings of this study is that human ZP retains its ability to bind spermatozoa, and to induce intraspermatic calcium influx and AR after fertilisation even if sperm penetration through fertilised ZP is strongly inhibited. The ZP-induced influx of calcium into human spermatozoa follows similar kinetics and has a similar sensitivity to VOCC inhibitors to that observed in response to solubilised ZP or purified ZP3 in mouse spermatozoa. The ZP bioactivity is not abolished by fertilisation, despite modification of the biochemical status of ZP2, from an uncleaved to a cleaved form. Compared to the mice model, this difference of ZP bioactivity could be linked to the presence of ZP4, which is not produced in mice. Thus, the place of ZP4 in the hZP matrix, its relation to other hZP glycoproteins, its function in the primary steps of gamete interaction (e.g. sperm binding/AR induction), and its modification after fertilisation require further study in humans.

## Methods

### Nomenclature used

We adopted the nomenclature recently proposed by Conner et al. [40], which follows the proposals of the NCBI committee. The four ZP genes are named *ZP1, ZP2, ZP3 *and *ZP4*, and encode proteins ZP1 to ZP4, respectively. The *ZP2, ZP3 *and *ZP4 *genes correspond to the *ZPA, ZPC *and *ZPB *genes, respectively, in Harris' nomenclature [41].

### Media

BWW medium contained 95 mM NaCl, 4.8 mM KCl, 1.7 mM CaCl_2_, 1.2 mM KH_2_PO_4_, 1.2 mM MgSO_4_, 5.6 mM glucose, 0.25 mM sodium pyruvate, 20 mM HEPES -free acid, 25 mM NaHCO_3_, 20 mM lactic acid, pH adjusted to 7.5 with NaOH. The osmolarity of this solution was 300–310 mosmol/l. In some experiments, a modified BWW medium devoid of Ca^2+ ^was used. This medium was prepared by omitting CaCl_2 _and adding 500 μM EGTA (Ca^2+ ^free BWW).

B2 medium was obtained from CCD (Paris, France) and Dulbecco's phosphate-buffered saline (PBS) was obtained from Sigma (Sigma-Aldrich, Saint-Quentin Fallavier, France).

### Products

Pimozide dissolved in absolute ethanol, Fura-2 AM dissolved in DMSO, sodium pyruvate, fluorescein isothiocyanate conjugated-goat anti-mouse antibody (FITC-GAM Ig), bovine serum albumin (BSA; fraction V) and HEPES were purchased from Sigma. Rhodamine-labelled *Pisum sativum *agglutinin (TRITC-PSA) was obtained from Vector (AbCys S.A., Paris, France). Anti-CD46 monoclonal antibodies were purchased from Immunotech (Marseille, France). All other chemicals (salts for buffers) were purchased from Merck/VWR (VWR, Fontenay-sous-Bois, France). Antibodies against human recombinant ZP2, produced in CHO cells, were a gift from J Harris (Zonagen, Inc., Woodlands, Texas, USA).

### Preparation of the gametes

#### - Spermatozoa

Human spermatozoa were obtained from healthy donors, as previously described [42]. Briefly, motile spermatozoa were selected by centrifuging semen samples through a two-phase Percoll gradient (47.5%–95%; 300 g, 20 min). The pellet of the 95% Percoll layer was washed by centrifugation in B2 medium (600 g, 10 min) and suspended at a concentration of 1 × 10^7^cells/ml in the same medium. The cells were incubated overnight at room temperature to allow a full capacitation of all sperm samples, knowing that a variability in the sperm capacitation time is particularly developed in humans [43]. For all sperm samples, motility was checked and found to exceed 70% after capacitation.

In some experiments, motile spermatozoa were divided into two aliquots after Percoll gradient selection, one of which was incubated in the presence and the other in the absence of 10 μM pimozide in B2 during capacitation.

#### - Oocytes and zona pellucida

Unfertilised and fertilised oocytes were collected after IVF or intracytoplasmic sperm injection (ICSI) at the Cochin-Saint Vincent de Paul Hospital (Paris, France). The unfertilised oocytes were at metaphase II (MII) and no signs of fertilisation (two pronuclei and GPII) or cleavage were observed 48 hours after insemination (IVF) or microinjection (ICSI). The fertilised oocytes consisted either of oocytes with more than two pronuclei visible 18 hours after fertilisation or of embryos seen at 2 pronuclei stage 18 hours after fertilisation and not transferred or frozen 48 hr after fertilisation due to high fragmentation rates (> 30%) or abnormal kinetics of cell division. Informed consent was obtained from each patient for the use of their fertilised or unfertilised oocytes for research in accordance with the guidelines of the Medical Ethics Committee of the Hospital.

These human oocytes were used either intact for functional assays or as a source of zona pellucida. In the case of oocytes recovered from IVF program, and before use, these oocytes were repetitively passed through a narrow pipette (internal diameter 125 μm) to dislodge the remaining spermatozoa bound to the ZP, if any. Human zona pellucida were mechanically isolated by disrupting oocytes or embryos incubated in distilled water and solubilising them by incubation in an acidic solution (5 mM NaH_2_PO_4_; pH 2.5) for 30 min at 37°C [22]. The acidic hZP extract was neutralised with an equal volume of 2 × BWW (pH 8.1) and used on the same day.

### Measurement of intracellular calcium concentration ([Ca^2+^]_i_)

The selected and capacitated motile spermatozoa (1 × 10^7^/ml) were incubated for 45 minutes at 37°C with 2 μM Fura-2 AM in 1% BSA in BWW, in a 5% CO_2_/95% air atmosphere. They were then washed by dilution (1/3-v/v) in BWW and centrifuged (600 g, 10 min). The pellets were suspended at a density of 1.5 × 10^7^cells/ml in BWW and kept at room temperature in the dark for a further 30 minutes before use. Aliquots (13 μl) of sperm suspension were placed in a glass microcuvette at 37°C and 4 μl of solubilised hZP or 5 mM NaH_2_PO_4 _buffered in 2 × BWW (control) were added. We used an hZP concentration of 2 hZP/μl, which lies within the range of concentrations used in most studies [30,32]. Fluorescence was recorded with a PTI M-2001 spectrofluorimeter (Kontron Instruments, Saint-Quentin-en-Yvelines, France) at an emission wavelength of 505 nm, using dual excitation at 340 and 380 nm (5 nm bandpass). Recordings were taken over 15 minutes and the sperm suspension was then collected and incubated for a further 15 min at 37°C in a 5% CO_2_/95% air atmosphere to allow completion of the AR.

We calibrated the fluorescent signal by carrying out parallel experiments in which sperm were permeabilised by incubation with 0.05% Triton X-100, to which 10 mM EGTA (pH 9.5) was subsequently added, for determination of the maximal and minimal fluorescence signals, respectively. [Ca^2+^]_i _was calculated from the 340/380 nm excitation ratio, as described by Grynkiewicz et al. [44] and reported by Yang et al. [45].

### Zona pellucida binding assay

Capacitated spermatozoa suspended in B2 medium were labelled with fluorescein isothiocyanate (FITC), as previously described [46], to distinguish them from spermatozoa eventually remaining bound to oocytes of IVF. Briefly, 100 μl of 1% FITC in 0.1 M KOH were diluted in 5 ml of PBS supplemented with 1% glucose. Capacitated spermatozoa were diluted 1/2 or 1/4 in the FITC solution and incubated for 10 min at 37°C then passed through a Percoll gradient in order to select only motile stained spermatozoa. We incubated 5 × 10^5 ^FITC-labelled capacitated spermatozoa with 500 μl of B2 medium containing 7 to 23 unfertilised or fertilised oocytes with an intact zona pellucida for 3 h (primary binding) or 18 h (secondary binding/penetration) at 37°C in an atmosphere containing 5% CO_2_/95% air.

For primary binding experiments, oocytes were washed in B2 medium by repeated aspiration in a glass pipette (internal diameter 250 μm), to dislodge loosely bound spermatozoa. The first aliquot of washed oocytes was placed in a glass depression slide and the number of FITC-labelled spermatozoa bound to each oocyte was determined with a Nikon E600 epifluorescence microscope (Nikon, St Quentin-en-Yvelines, France). The second aliquot of washed oocytes was treated as described below to determine the acrosomal status of bound spermatozoa.

After 18 hours of incubation time, the oocytes were manipulated according to the procedure reported by Liu and Baker [47] to assess the sperm-zona pellucida penetration. Briefly, the oocytes were washed to remove loosely bound spermatozoa and repetitively aspirated in and out of a pipette with an internal diameter of 125 μm to dislodge spermatozoa bound to the ZP, which were then examined for AR. These oocytes, cleared of spermatozoa on the surface of the ZP, were placed on a slide in a 20 μl drop of 0.2% BSA in PBS and the number of FITC-labelled spermatozoa remaining embedded within the zona pellucida or present in the perivitelline space were counted under the epifluorescence microscope at × 400 magnification.

### Measurement of the acrosome reaction

#### - After induction by solubilised human zona pellucida

At the end of each incubation with solubilised hZP or control medium, spermatozoa were harvested by centrifugation (600 g, 5 min) and suspended in 100 μl of 1% BSA in PBS supplemented with anti-CD46 antibody (10 μg/ml). CD46 is an antigen located on the internal acrosomal membrane and is exposed only after completion of the AR [48]. After incubation for 30 min at 37°C, the sperm suspension was incubated for a further 30 min with FITC-GAM IgG (1/100). The spermatozoa were then washed twice in 1% BSA in PBS and stored in 0.1% formaldehyde in PBS for up to 24 hours before flow cytometry analysis (FACS Calibur, Becton Dickinson, Pont-de-Claix, France). The zona pellucida-induced AR (ZPIAR) was quantified on at least 3000 cells and the results are expressed as the difference between the AR promoted by hZP and the spontaneous AR observed in controls.

#### - After induction by intact human egg zona

After 3 or 18 h of incubation with oocytes or embryos, as described above in the "Zona pellucida binding assay" section, spermatozoa firmly bound to the ZP were removed in 20 μl drops of 0.2% BSA in PBS by repeated aspiration through a pipette with an internal diameter (125 μm) slightly smaller than the diameter of the oocyte. The acrosomal status of these spermatozoa was then determined using a modified version of the method of Cross et al. [49]. Briefly, spermatozoa were air-dried on slides, fixed in ethanol for 30 minutes at +4°C and stained with TRITC-PSA (25 μg/ml in PBS) for 15 minutes. They were then washed in distilled water and mounted in Cityfluor (VWR, Fontenay-sous-Bois, France). When the AR rate was assessed in the incubation medium around the oocytes, 200 cells were examined for their acrosomal status. For the spermatozoa bound to ZP, from 50 to 250 cells (majority between 100–150 cells) were examined according to the experiments. Spermatozoa were examined under an epifluorescence microscope, at a magnification of × 1000. Spermatozoa with a fluorescent band at the equatorial segment or with no staining of the head were considered to have undergone the AR. As only motile sperm were able to bind to the ZP, we carried out no viability staining.

### Electrophoretic separation and immunoblot analysis

Isolated zonae pellucidae were solubilised (v/v) in reducing sample buffer (20% glycerol, 4% SDS, 0.125 M Tris-HCl pH 6.5, 10% β-ME), heated for 5 min at 96°C and centrifuged for 5 min at 12000 g. The supernatant (equivalent of 3 solubilised ZP) was subjected to electrophoresis at 150 V for 45 min. One dimensional SDS-PAGE was performed with a 7% acrylamide gel, using a Mini PROTEAN II Cell Apparatus (Bio-Rad Laboratories, Marne-la-Coquette, France).

The separated proteins were electrotransferred onto PVDF membranes (GE Healthcare Bio-Sciences, France) for ZP2 detection. The blots were blocked by incubation with Tris buffer containing 1% (w/v) gelatin and 0.1% (v/v) Tween-20 and were then incubated with anti-rhZP2 antibodies diluted 1:2000 in the same buffer. Antibody binding was detected by incubation with horseradish peroxidase-conjugated anti-IgG antibody (dilution 1:30000), by enhanced chemiluminescence (ECL+; GE Healthcare Bio-Sciences).

### Statistical analysis

The data are expressed as means ± SEM and were compared using Student's t test or the Mann and Whitney test. For the AR and penetration rates, chi^2 ^tests were used for comparisons. Values of p < 0.05 were considered statistically significant.

## Authors' contributions

CP participated in the design of the study, carried out the spectrofluorimetry studies, the AR measurements and the redaction of the manuscript. JA carried out the electrophoretic studies and the binding tests and helped to draft the manuscript. PF and RL participated in the oocyte collection and the preparation of the zona pellucida. PJ participated in the design of the study and the final version of the manuscript. CS conceived the study and helped for several experiences and for redaction of the manuscript. All these authors had several discussions concerning the study, read and approved the final manuscript.
